# Whole Genome Sequencing of Influenza A and B Viruses With the MinION Sequencer in the Clinical Setting: A Pilot Study

**DOI:** 10.3389/fmicb.2018.02748

**Published:** 2018-11-13

**Authors:** Kazuo Imai, Kaku Tamura, Tomomi Tanigaki, Mari Takizawa, Eiko Nakayama, Takahiko Taniguchi, Misako Okamoto, Yasumasa Nishiyama, Norihito Tarumoto, Kotaro Mitsutake, Takashi Murakami, Shigefumi Maesaki, Takuya Maeda

**Affiliations:** ^1^Department of Infectious Disease and Infection Control, Saitama Medical University, Saitama, Japan; ^2^Center for Clinical Infectious Diseases and Research, Saitama Medical University, Saitama, Japan; ^3^Department of Infectious Diseases, Self-Defense Forces Central Hospital, Japan Ground Self-Defense Forces, Tokyo, Japan; ^4^NBC Counter Medical Unit, Japan Ground Self-Defense Forces, Tokyo, Japan; ^5^Camp Asaka Medical Office, Japan Ground Self-Defense Forces, Tokyo, Japan; ^6^Department of Infectious Disease and Infection Control, Saitama Medical University International Medical Center, Saitama, Japan; ^7^Department of Microbiology, Saitama Medical University, Saitama, Japan

**Keywords:** influenza virus, MinION, MiSeq, whole genome sequencing, Japan

## Abstract

**Introduction:** Whole genome sequencing (WGS) of influenza viruses is important for preparing vaccines and coping with newly emerging viruses. However, WGS is difficult to perform using conventional next-generation sequencers in developing countries, where facilities are often inadequate. In this study, we developed a high-throughput WGS method for influenza viruses in clinical specimens with the MinION portable sequencer.

**Methods:** Whole genomes of influenza A and B viruses were amplified by multiplex RT-PCR from 13 clinical specimens collected in Tokyo, Japan. Barcode tags for multiplex MinION sequencing were added with each multiplex RT-PCR amplicon by nested PCR with custom barcoded primers. All barcoded amplicons were mixed and multiplex sequencing using the MinION sequencer with 1D^2^ sequencing kit. In addition, multiplex RT-PCR amplicons generated from each clinical specimen were sequenced using the Illumina MiSeq platform to validate the performance of MinION sequencer. The accuracy, recall, and precision rates of MinION sequencing were calculated by comparing the results of variant calling in the Illumina MiSeq platform and MinION sequencer.

**Results:** Whole genomes of influenza A and B viruses were successfully amplified by multiplex RT-PCR from 13 clinical samples. We identified 6 samples as influenza type A virus H3N2 subtype and 7 as influenza B virus Yamagata lineage using the Illumina MiSeq platform. The overall accuracy, recall, and precision rates of the MinION sequencer were, respectively 99.95%, 89.41%, and 97.88% from 1D reads and 99.97%, 93.28%, and 99.86% from 1D^2^ reads.

**Conclusion:** We developed a novel WGS method for influenza A and B viruses. It is necessary to improve read accuracy and analytical tools in order to better utilize the MinION sequencer for real-time monitoring of genetic rearrangements and for evaluation of newly emerging viruses.

## Introduction

Influenza viruses, which are enveloped, single-stranded negative-sense RNA viruses, belong to the *Orthomyxoviridae* family and cause respiratory diseases in humans around the world. While there are four types of seasonal influenza viruses – types A, B, C, and *Thogotovirus* – influenza A and B viruses in particular can cause seasonal epidemics that have high rates of morbidity and mortality (Taubenberger and Morens, 2008). The genomes of influenza A and B viruses are composed of the following eight RNA segments: segment 1, RNA polymerase PB2 unit; segment 2, RNA polymerase PB1 unit; segment 3, RNA polymerase PA unit; segment 4, hemagglutinin (HA); segment 5, nucleoprotein (NP); segment 6, neuraminidase (NA); segment 7, matrix (M); and segment 8, non-structural protein (NS).

The genomes of influenza viruses frequently develop genetic changes and have a high level of genetic diversity, which contributes to their ability to evade the human immune system (Grenfell et al., 2004). To cope with the emergence of new influenza virus strains and select appropriate vaccine strains, considerable effort is being devoted to monitoring the genetic rearrangements of these viruses (Ghedin et al., 2005; Shu and McCauley, 2017). A simple amplification method using RT-PCR for influenza virus has been developed and is contributing to the efficient surveillance of influenza viruses via next-generation sequencing (NGS) (Zhou et al., 2009; Zhou et al., 2014; Zhao et al., 2016). NGS technology is used in a wide range of applications, including population surveillance for emerging strains and the subsequent creation of new vaccines, outbreak research in communities, and tracking of nosocomial infections (Briand et al., 2017; Harvala et al., 2017; Houlihan et al., 2018; MacFadden et al., 2018). However, amplicon sequencing using NGS platforms such as 454 (Roche Life Science, Penzberg, Germany), the Illumina platform (Illumina, San Diego, CA, United States), and Ion Torrent technology (Thermo Fisher Scientific, Waltham, MA, United States) has high initial and running costs and is time-consuming. Therefore, it is challenging to establish facilities with an NGS platform for the surveillance of influenza viruses in developing countries (Vemula et al., 2016).

The MinION nanopore sequencer, recently developed by Oxford Nanopore Technologies (ONT; Oxford, United Kingdom), is a palm-size portable sequencer that can read long-read single DNA molecules in real time and is regarded as third-generation DNA sequencing technology. The most notable feature of the MinION sequencer is its portability, which makes it different from other NGS platforms. For this reason, the MinION sequencer has been used as an alternative means of whole genome sequencing (WGS) and can be used for genomic surveillance and infection control for certain pathogens, taking advantage of its high portability, and its quick and easy sample preparation. However, its high sequence error rate remains a challenge (Quick et al., 2015, 2016; Hoenen et al., 2016). In 2017, ONT released the new 1D^2^ method, which has higher single-read accuracy than the standard 1D method^1^. The WGS method for influenza A virus using the MinION sequencer has been previously reported (Wang et al., 2015). However, the method still has some practical problems, such as a high per-sample cost because of the single plex sequencing, applicability to only influenza A virus, and unknown performance in clinical settings.

In this study, we developed, a quick and accurate multiplex WGS method for influenza A and B viruses using clinical specimens, the MinION nanopore sequencer, and the 1D^2^ method.

## Materials and Methods

### Clinical Samples and Ethics

Clinical samples were collected from suspected influenza patients at the Japan Self-Defense Forces Central Hospital and Medical Office of Camp Asaka, Tokyo, Japan, from December 2017 to April 2018. All patients were diagnosed based on commercially available rapid diagnostic tests for influenza using nasopharyngeal swab specimens, according to the manufacturer’s instructions (QuickNavi-Flu 2; Denka Seiken, Niigata, Japan). In particular, the samples obtained were composed of two specimens, which were separated for subsequent tests. One swab was used for the rapid diagnosis of influenza and the other for the subsequent RNA extraction of influenza virus. Each swab specimen was stored at -80°C until just before use. A diagnosis of influenza was ultimately confirmed via RT-PCR according to previously reported protocols for influenza viruses A (M30F2/08 and M264R3/08 for all subtypes) and B (Bvf224 and Bvr507 for Victoria lineage, and Bvf226 and BYr613 for Yamagata lineage). Both primer sets are recommended for the detection of influenza viruses by the World Health Organization [World Health Organization (WHO), 2017]. To evaluate our procedures in clinical practice, 13 nasopharyngeal swab samples were confirmed as influenza-positive (6 = influenza A virus and 7 = influenza B virus Yamagata lineage) and used in our study.

This study was conducted in accordance with the Declaration of Helsinki and was approved by the Institutional Ethics Committee of the Self-Defense Forces Central Hospital (No. 29-012). All samples were collected after written informed consent was obtained.

### RNA Extraction and DNase Treatment

Total RNA was extracted using a High Pure Viral RNA Kit (Roche Life Science) according to the manufacturer’s instructions. Additionally, each eluted RNA was treated with 4U of TURBO DNase (Thermo Fisher Scientific) for 30 min at 37°C to remove the human genomic DNA and purified again by Agencourt RNAClean XP (Beckman Coulter, Brea, CA, United States) according to the manufacturer’s instructions.

### Multiplex RT- PCR and Purification

Supernatants of each purified RNA sample (5 μL) were then used for multiplex RT-PCR reactions. In this scheme, viral RNA segments of influenza A (PB2 = 2.3 kb, PB1 = 2.3 kb, PA = 2.2 kb, HA = 1.8 kb, NP = 1.6 kb, NA = 1.4 kb, M = 1.0 kb, and NS = 0.9 kb) were simultaneously amplified using two primers (Uni12, Uni13; Supplementary Table S1) targeting the highly conserved sequence of viral RNA termini, which are commonly present at the ends of the PB2, PB1, PA, HA, NP, NA, M, and NS genome segments, following protocols previously reported for influenza A virus (Zhou et al., 2009). For influenza B (PB2 = 2.3 kb, PB1 = 2.3 kb, PA = 2.3 kb, HA = 1.8 kb, NP = 1.8 kb, NA = 1.5 kb, M = 1.1 kb, and NS = 1.0 kb), multiplex amplification was simultaneously performed with multiple primer sets targeting the corresponding conserved sequence of each viral RNA termini in accordance with a previous report (Supplementary Table S1; Zhou et al., 2014). Multiplex RT-PCR amplicons were generated by the SuperScript^TM^ III One-Step RT-PCR System with Platinum^TM^ Taq High Fidelity DNA Polymerase (Thermo Fisher Scientific). PCR products were analyzed using the Agilent 2100 Bioanalyzer (Agilent Technologies, Santa Clara, CA, United States) with an Agilent DNA 7500 Kit (Agilent Technologies). Each PCR product was purified again using Agencourt AMPure XP according to the manufacturer’s instructions.

### Sequencing of Multiplex RT-PCR Products Using the Illumina MiSeq Sequencer

The complete influenza virus genome was analyzed with multiplex RT-PCR amplicon sequencing using the Illumina MiSeq platform. Paired-end libraries for MiSeq platform were prepared using Nextera XT DNA Library Prep Kit (Illumina) and Nextera XT Index Kit (Illumina), and sequencing was performed using a 300-cycle (2 × 150-bp paired-end) MiSeq v2 reagent kit (Illumina) via MiSeq platform according to the manufacturer’s protocols.

### Analysis of Illumina MiSeq Platform Data

Low-quality reads in demultiplexed data were removed and 3′ terminal nucleotides were trimmed with SeqKit and mapped with BWA-MEM (v 0.7.15) using the reference sequences for influenza A (GenBank; CY259943-50) and influenza B viruses (GenBank; MH233733-40). Single nucleotide variant calling from mapped data was obtained by Samtools, Picard, and GATK via “HaplotypeCaller” commands (Cornish and Guda, 2015).

### Sequencing of PCR Products Using the MinION Sequencer

To enable use of multiplex MinION sequencing at reduced cost with shorter analysis times, each specific barcode tag for the MinION was added at the start of each reverse primer for eight viral RNA segments (influenza A, Uni13/Inf1-Bc; influenza B, UniR-Bc) (Supplementary Table S1). An additional nested PCR assay with custom barcoded primers was performed consecutively to match the sequence reads to the correct clinical specimens. Briefly, 1 μL of 10 ng from each purified PCR amplicon of eight viral RNA segments was used as template DNA. The nested PCR assays were performed using KAPA HiFi HotStart ReadyMix PCR Kit (Kapa Biosystems, Wilmington, MA, United States) and thermal cycling was carried out under the following conditions: 94°C for 5 min, followed by 18 cycles at 98°C for 10 s, 60°C for 30 s, 72°C for 120 s, and a final extension at 72°C for 5 min. The nested PCR products with each ONT-specific barcode tag were analyzed using the Agilent 2100 Bioanalyzer with an Agilent DNA 7500 Kit. Each PCR product was consequently re-purified with Agencourt AMPure XP according to the manufacturer’s instructions. The nested PCR amplicons (300 ng each) were measured by Qubit 3.0 Fluorometer (Thermo Fisher Scientific) and simultaneously processed for library preparation using the SQK-LSK308 1D^2^ Sequencing Kit (ONT) according to the manufacturer’s instructions. After the MinION Platform QC run, the mixed DNA library derived from 13 clinical samples was loaded into a single MinION Flow Cell (FLO-MIN107 R9.5 Version) and the “NC_48Hr_sequencing_FLO-MIN107_SQK-LSK308_plus_basecaller” protocol was initiated using MinKNOW software (v 1.4.1, ONT).

### Analysis of MinION Data

Local basecalling was performed using MinKNOW automatically in real time. 1D^2^ reads were generated from FAST5 reads by Albacore (v 2.2.1). Low-quality reads and < 500- and > 3000-length reads were removed by SeqKit (v 0.8.1). Demultiplexing and adapter trimming was performed using Porechop (v 0.2.2), and all ONT-barcode data were eventually collected and mapped by Minimap2 (v 0.7.15) using the reference sequences for influenza A H3N2 (GenBank; CY259943-50) and influenza B Yamagata lineage (GenBank; MH233733-40). Single nucleotide variant calling from mapped data was obtained by Samtools and BCFtools (v 1.5.0) via the mpileup command, and variant calling under 10× read depth was filtered. Mapped data were visualized by IGV software (v 2.3.8) and analyzed by Samtools and Qualimap (v 2.2.1).

## Results

### Multiplex RT-PCR for Clinical Samples

All eight segments of influenza gene from each nasopharyngeal swab samples were successfully amplified with multiplex RT-PCR. Based on the results of RT-PCR amplicon sequencing using the Illumina MiSeq, 6 samples were identified as influenza A H3N2 subtype and 7 samples were identified as influenza B Yamagata lineage. The complete influenza virus genomes were deposited in the Global Initiative on Sharing All Influenza Data database (GISAID: EPI_ISL_313902 - 313914).

### Results of MinION Sequencing, Mapped for Reference Genomes

**FIGURE 1:**
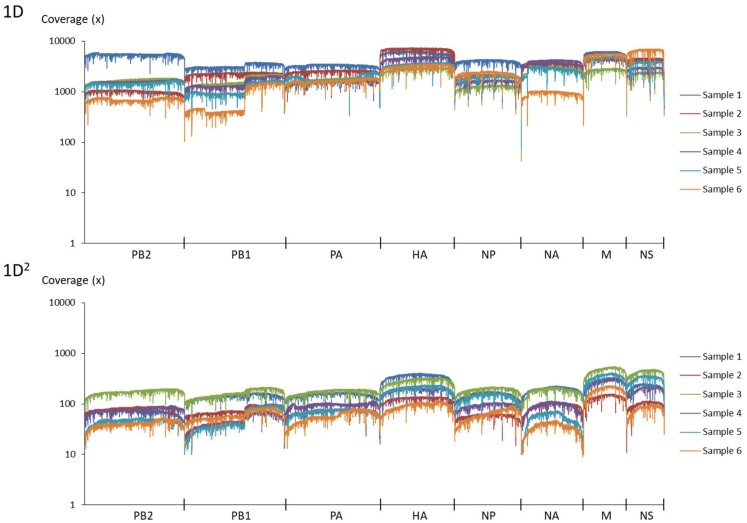
Coverage of MinION sequencing reads for a reference genome of influenza A virus. The upper graph shows the results of 1D reads and the lower graph shows results of 1D^2^ reads.

**FIGURE 2:**
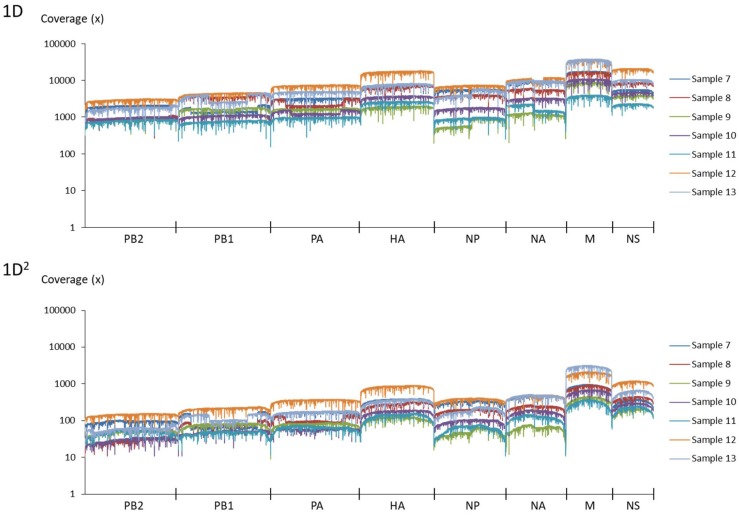
Coverage of MinION sequencing reads for a reference genome of influenza B virus. The upper graph shows the results of 1D reads and the lower graph shows results of 1D^2^ reads.

Amplicons with ONT-specific barcodes were successfully amplified by nested RT-PCR. Finally, a total of 2,120,657 1D reads (total 3.2 Gbases) were generated within 48 h after the start of MinION sequencing, and a total of 71,066 1D^2^ reads (total 101 Mbases) were generated by Albacore. Among the total collected reads, 1.87% ± 1.42% and 5.97% ± 2.83% were classified into ONT-barcodes after barcode demultiplexing as 1D and 1D^2^, respectively. Tables 1, 2, and Supplementary Table S2 show the results of mapped data for reference sequences by Minimap2, and Figures 1, 2 show the depth of coverage for the reference sequences per sample. The mean coverage per sample for influenza A virus was 1,578 and 89 and that for influenza B virus was 6,545 and 86 for 1D and 1D^2^, respectively. The raw sequence data of the Illumina MiSeq and MinION sequencers were deposited in the Sequence Read Archive (SRA: PRJNA491222).

**Table 1:** Results of whole genome sequencing of influenza A virus using the MinION sequencer.

Influenza A virus (*n* = 6)						
Segment of influenza A virus	Gene length (bp)	Chemistry	Mean no. of mapped reads	Mean no. of mapped bases (bp)	Mean coverage	Accuracy rate (%)
PB2	2,316	1D	1,988	3,654,198	1,878	99.94
		1D^2^	115	205,455	98	99.98
PB1	2,316	1D	2,723	4,348,554	2,303	99.92
		1D^2^	146	226,946	113	99.94
PA	2,209	1D	3,056	5,087,913	4,226	99.94
		1D^2^	154	249,691	228	99,99
HA	1,737	1D	4,764	7,340,349	2,384	99.90
		1D^2^	271	395,417	127	99.99
NP	1,542	1D	2,716	3,676,187	2,861	99.93
		1D^2^	158	195,941	117	99.97
NA	1,441	1D	3,320	4,121,981	5,497	99.94
		1D^2^	144	168,456	303	99.97
M	1,002	1D	5,889	5,508,393	2,189	99.98
		1D^2^	362	303,262	111	100
NS	865	1D	5,137	4,081,978	2,816	100
		1D^2^	273	206,650	145	100
Total	13,428	1D	38,548	37,819,556	1,578	99.94
		1D^2^	3,568	1,951,821	89	99.98


**Table 2:** Results of whole genome sequencing of influenza B virus by MinION sequencer.

Influenza B virus (*n* = 7)						
Segment of influenza B virus	Gene length (bp)	Chemistry	Mean no. of mapped reads	Mean no. of mapped bases (bp)	Mean coverage	Accuracy rate (%)
PB2	2,367	1D	1,830	6,919,598	1,839	99.93
		1D^2^	96	2,957	133	99.98
PB1	2,340	1D	3,416	9,344,993	2,957	100
		1D^2^	182	4,108	203	100
PA	2,275	1D	3,974	16,632,486	4,108	99.95
		1D^2^	203	8,976	462	99,99
HA	1,853	1D	7,614	8,110,509	8,976	99.98
		1D^2^	416	4,466	248	99.99
NP	1,816	1D	4,172	11,079,003	4,466	99.97
		1D^2^	241	7,241	350	99.97
NA	1,530	1D	6,896	26,258,563	7,241	99.93
		1D^2^	377	22,735	1,472	99.97
M	1,155	1D	19,491	11,531,285	22,735	99.89
		1D^2^	1,364	10,858	628	100
NS	1,026	1D	8,533	94,229,268	10,858	99.96
		1D^2^	560	6,545	360	100
Total	14,362	1D	62,535	4,352,833	6,545	99.95
		1D^2^	4,973	1,839	86	99.98


### Comparison With the Illumina MiSeq Platform

Tables 1, 2, and Supplementary Table S3 show the nucleotide sequence identity between influenza gene segments sequenced using the MinION and Illumina MiSeq sequencers. The total accuracy rate of nucleotide sequence identity for influenza A virus when using the MinION sequencer was 99.94 and 99.98% and that for influenza B virus was 99.95 and 99.98% for 1D and 1D^2^, respectively. Variant calling from 1D^2^ rather than from 1D reads decreased the numbers of false positives and false negatives when using the MinION sequencer. The total recall and precision rates were 89.41% and 97.88% from 1D reads and 93.28 and 99.86% from 1D^2^ reads, respectively (Supplementary Table S4). The accuracy of the read level of MinION sequencing, which was calculated based on the mapped data for consensus influenza genome sequences generated from Illumina MiSeq reads, was 88.74% for 1D reads and 92.81% for 1D^2^ reads (Supplementary Table S5).

## Discussion

In this pilot study, we proposed a high-throughput MinION sequencing protocol that can be performed both in the field and in the clinical setting. We demonstrated that multiplex MinION sequencing could achieve variant calling easily and rapidly from 1D^2^ reads generated by combining multiplex RT-PCR methods for both influenza A and B viruses. In particular, sample processing and library preparation processes can be completed within 12 h without expensive or large experimental devices. Furthermore, the sequencing data can be analyzed in real time using a laptop computer. Moreover, samples can be prepared and sequenced without storage or transfer to a fully equipped laboratory. Therefore, our method would have the potential to allow real-time monitoring of both genetic rearrangements and the emergence of new influenza virus strains.

For accurate surveillance and vaccine subpopulation selection, a highly precise sequencing and variant calling method is always required. In particular, the recall and precision rate of the current MinION sequence technology were inferior to those of the Illumina MiSeq platform, and these limitations were similar to findings from other studies on genotyping using the MinION sequencer (Wang et al., 2015; Cornelis et al., 2017; Lang et al., 2018; Runtuwene et al., 2018). Thus, our method is currently unsuitable to replace the Illumina platform. The high error rate and number of coverages of the MinION sequencer could affect the accuracy of variant calling because the read level accuracy rate was estimated to be about 88.74% for 1D and 92.81% for 1D^2^. In fact, false-positive results can be significantly reduced via high-quality 1D^2^ reads (precision rate: 97.88% from 1D reads vs. 99.86% from 1D^2^ reads). However, 1D^2^ reads would unfortunately still include false negatives due to single nucleotide polymorphisms (recall rate: 89.41% from 1D reads vs. 93.28% from 1D^2^ reads) because they would include deletion errors more frequently around the homopolymer sequence and have lower coverage than 1D reads. Further improvements in sequence accuracy of the MinION sequencer would make it possible to perform surveillance and vaccine subpopulation selection.

In this study, we used the clinically applicable amplicon sequencing protocol, to analyze influenza virus genome sequencing. Previously, Greninger et al. (2015) demonstrated that metagenomic sequencing analysis with the MinION sequencer could detect unknown emerging viruses without prior information and would be desirable for point-of-care genomic sequencing even in the developing world. However, its sequencing scheme still has lower sensitivity and specificity levels compared with the amplicon sequencing protocol of clinical samples, because of contamination from the large amount of host genome fragments in clinical samples (Rosseel et al., 2012; Quick et al., 2017). Moreover, there is currently no practical preparation kit for adding barcode tags in multiple-sample processing based on metagenomic sequencing with the 1D^2^ sequencing kit, even though multiplex sequencing is an essential technology that reduces the cost and time of analysis. Amplicon sequencing can solve one of these problems precisely by reducing contamination from host genome fragments via the added multiplex RT-PCR step. Furthermore, it is possible to simply add a barcode tag using nested PCR, thus enabling multiple sample processing. Of course, the DNA fragmentation step, including mechanical or chemical pretreatment, is required for the Illumina MiSeq platform because of its read length of up to 600 bp. However, a fragmentation step and additional device for amplicon sequencing are not necessary when using the MinION sequencer. This is because it can obtain long read data over 100 kb, which is another one of its advantageous characteristics (Jain et al., 2018). Unfortunately, even with high-fidelity DNA polymerase reactions, amplification by multiple RT-PCR and nested PCR could be a drawback as it may cause PCR-generated errors (Brandariz-Fontes et al., 2015; Potapov and Ong, 2017).

Multiplex sequencing with introduced barcode tag nucleotides would also contribute to improving the accuracy of pairing 1D sequence reads. In this study, we used nested PCR protocols to add 30-base ONT-specific barcodes against multiplex RT-PCR products of eight viral RNA segments and analyzed multiple samples simultaneously. In fact, in 1D^2^ sequencing using PCR amplicons, false-positive pairings between different samples could occur because 1D^2^ reads are generated with aberrant *in silico* chimeric reads from 1D reads. However, the addition of 30-base ONT-specific barcodes can effectively decrease the risk of false 1D^2^ pairing of strands.

Avian influenza types, such as influenza A virus subtypes H5N1 and H7N9, are likely to emerge with the potential to cause pandemics in developing countries (Oshitani et al., 2008; Fedson, 2009). Furthermore, recent meta-analyses showed that morbidity and mortality are higher in patients with influenza viruses in developing countries than in those in developed countries (Nair et al., 2011; Ortiz et al., 2013). The WHO indicated an urgent need for strengthening local responses to emerging influenza viruses [World Health Organization (WHO), 2005]. Whole genome analyses of influenza viruses using NGS is important for the surveillance of novel strains of influenza virus with genetic rearrangements and antiviral resistance. Compared with other NGS technologies, the MinION sequencer has excellent portability in addition to reduced initial installation costs, running costs, and analysis times. In the near future, the low-priced Flongle Flow Cell sequencing device^2^ – an adapter for MinION flow cells that has 126 channels compared with 512 channels in the current MinION – will become available and provide further versatility. Therefore, this novel procedure has the potential to be applicable as a surveillance technology for emerging influenza strains and for use even in situations where research facilities are inadequate.

## Conclusion

We developed novel high-throughput MinION sequencing methods for real-time sequencing of influenza A and B viruses using a portable sequencing setup with quick and easy sample preparation. Further improvement of read level accuracy and analytical tools is necessary to achieve better utilization of the MinION sequencer for both real-time monitoring of genetic rearrangements and evaluation of newly emerging viruses.

## Author Contributions

KT, YN, MO, and TMa designed the research. KI, ToT, MT, EN, TaT, and NT performed the research. KM, SM, and TMu provided scientific guidance. KI and TMa prepared the manuscript.

## Conflict of Interest Statement

The authors declare that the research was conducted in the absence of any commercial or financial relationships that could be construed as a potential conflict of interest.

## Footnotes

1 https://nanoporetech.com/

2 https://nanoporetech.com/products/flongle
